# Electrical Activity of the Anterior Temporal and Masseter Muscles in Mouth and

**DOI:** 10.1016/S1808-8694(15)30608-X

**Published:** 2015-10-18

**Authors:** Aline Ferla, Ana Maria Toniolo da Silva, Eliane Castilhos Rodrigues Corrêa

**Affiliations:** 1 MSc in Human Communication Disorders at UFSM, Speech and Hearing Therapist at the Municipal Clinic of Novo Hamburgo-RS.; 2 PhD in Human Communication Disorders at the Federal University of São Paulo, Speech and Hearing Therapist, Adjunct Professor at UFSM.; 3 PhD in Oral-Dental Biology at the Campinas State University, Physical Therapist, Adjunct Professor at UFSM. Federal University of Santa Maria.

**Keywords:** children, electromyography, mouth breathing

## Abstract

Mouth breathing has been associated with severe impact on the development of the stomatognathic system.

**Aim:**

This paper aims to analyze the electromyographical findings and patterns of electrical activity of the anterior temporal and masseter muscles in mouth and nasal breathing children.

**Materials And Method:**

The patients were divided into two groups: mouth breathers (n=17) and nasal breathers (n=12). The children underwent bilateral electromyographic examination of the anterior temporal and masseter muscles at maximal intercuspal position and during usual mastication. A Myosystem Br-1 electromyograph with 12 acquisition channels, amplification with total gain of 5938, rate of acquisition of 4000 Hz, and band-pass filter of 20-1000Hz, was used in the examination. The signal was processed in Root Mean Square(RMS), measured in µV, analyzed and expressed as a normalized percentage. The data set was statistically treated with the T-test (Student).

**Results:**

The observed level of electrical activity in the mouth breathing (MB) group was lower in all analyzed muscles, with statistical significance found only in the left temporal muscle; during mastication, mouth breathers also presented increased electrical activity on the right side and on the temporal muscle.

**Conclusion:**

Mouth breathing impacts the electrical activity of the muscles studied at maximal intercuspal position and during usual mastication.

## INTRODUCTION

Breathing is a vital function throughout our lives, and has direct impact on the maintenance of the skeletal, dental, and muscle organization of the stomatognathic system. The literature indicates that when inadequate respiratory patterns manifest themselves in the form of mouth breathing, many are the associated alterations. Postural compensations such as head extension to facilitate the passing of air^1^, ^2^, ^3^, ^4^, ^5^, ^6^, predominantly dolicofacial growth^7^, ^8^, ^9^ and changes in mastication^10^, ^11^, ^12^, ^13^, among others, have been observed in mouth breathers and noted as characteristic features of this alteration.

Although mouth breathing has been extensively researched, very little has been discussed as to how mastication develops in mouth breathers. It is known that mastication can be affected by factors such as teeth and temporomandibular joint status, occlusion type, and head and neck posture^14^, ^15^, ^16^, ^17^, ^18^. Some have considered, however, that mastication is also impacted by predominantly vertical facial growth patterns and mouth breathing, mainly due to inadequate eating habits.

Speech therapists have noted that most mouth breathers present altered mastication. However, masticatory muscle function is most frequently assessed in a clinical practice setting, where subjective evaluations often lead to different opinions among care professionals.

Electromyography can be used to assist in the assessment and diagnosis of such patients, and to analyze muscle electrical activity in an objective manner, as it has been used for years in speech therapy research. The electromyograph captures and amplifies action potentials of voluntary muscle contraction, providing additional diagnostic aid in evaluating neuromuscular system status^19^. Careful clinical examination is indispensable, and electromyography - if used judiciously - may assist in the comprehension of the electrical activity patterns of face and mastication muscles, providing care professionals with a more objective diagnosis and consequently a more effective approach to oral motor disorders.

## MATERIALS AND METHOD

### I. Research characterization

This research project was approved by the Research Ethics Committee of our institution under permit 105/2003. This is a qualitative, quantitative, cross-sectional, on-field study. It attempted to analyze, using electromyography, the electric activity patterns of the anterior temporal and masseter muscles bilaterally in mouth-breathing children and to compare their results against those of nasal breathing children in maximal intercuspal position and during normal mastication.

### II. Group selection procedure

Forty-five children were initially picked. The following procedures were performed for all of them: signature of a free informed consent form by their parents or caretakers, interview, ENT assessment, speech evaluation, and dental review. A multidisciplinary approach was adopted in patient screening and diagnose as, according to the literature, many are the alterations associated with mouth breathing^8^, ^11^, ^20^, ^21^, ^22^, ^23^, ^24^.

### III. Group selection criteria

After the above-mentioned procedures were completed, patients were split into mouth breathing or nasal breathing groups, based on the criteria described below:

Mouth breathing group: information was gathered from parents as to the presence of respiratory problems; presence of alterations, under speech evaluation, indicative of mixed or predominantly mouth breathing; ENT diagnosis of mixed or predominantly mouth breathing.

Nasal breathing group: absence of complaints about respiratory problems; absence of alterations under speech evaluation indicative of mixed or predominantly mouth breathing.

The following were also considered as criteria when assigning patients to groups: not having speech or dental treatment during the course of the study or being treated previously; absence of signs and/or symptoms indicative of TMJ disorder; absence of open bite and/or cross-bite occlusion; absence of signs indicative of neurological involvement.

Occlusion types were not considered in patient group assignment. A statistical analysis was performed using the ANOVA test to verify the correlation between occlusion type and electromyographic findings. The analysis was carried out to eliminate the interference of variable ’occlusion type’ in data analysis, but results pointed to no statistically significant difference among patient subgroups.

### IV. Group characterization

Sixteen children were excluded from the original group of forty-five, as they did not meet the study admission criteria. The groups were therefore categorized the following way:

a) Mouth breathing (MB): 17 children, 7 girls and 10 boys, with ages ranging from 8 years and 11 months to 11 years and 8 months.
b) Nasal breathing (NB): 12 children, 8 girls and 4 boys, with ages ranging from 9 years and 5 months to 12 years and 11 months.

### V. Electromyographic evaluation

Muscle activity assessment was based on the bilateral electromyographic records of the right anterior temporal (RT), left anterior temporal (LT), right masseter (RM), and left masseter (LM) muscles at maximal intercuspal position and under mastication (tests 1 and 2). Three data collection sessions were held for each test. Before acquiring the electromyographic data, the children were trained to ensure result consistency. The skin on the region of the muscles to be studied was cleaned with cotton balls drenched in ethanol. A gel-covered reference electrode was placed in one of the patients’ forearms to avoid electromagnetic interference during examination and to protect the children. During the examination the patients were comfortably seated on a chair, with their backs in the upright position, feet on the floor, and heads positioned according to Frankfurt's horizontal plane, parallel to the ground.

Test 01 - maximal intercuspal position - patients were told to clench their jaws, contracting their masticatory muscles bilaterally and simultaneously, and to remain at maximal intercuspal position for 5 seconds. They were given the following verbal command: 'squeeze it, squeeze it, squeeze it...’.

Test 02 - usual mastication - children were initially told to chew some Trident gum for an average of 15 seconds so as to achieve consistent results before the data was recorded. This particular chewing gum was picked as it is easy to handle and for its acceptance among the children. The patients were requested to chew normally, however at a pace set by the researcher clapping her hands for 10 seconds.

A Myosystem Br-1 electromyograph made by PROSECON Ltda was used in the examination. It has twelve channels, eight of which used for EMG data acquisition and four to capture auxiliary data, and a 12-bit resolution A/D converter. The device was designed in accordance with international regulations and its calibration was done as per standard specifications^25^. In this study the adopted channel sampling frequency was 4000 Hz; high-pass filter of 20 Hz and low-pass of 1000 Hz; total gain of 5938.

Four input channels were used to acquire electromyographic records, one for each of the muscles studied. Four single differential surface active electrodes were used. Each consisted of two rectangular (10x2mm) silver (Ag) plates spaced 10mm from one another attached to an acrylic resin capsule (23x21x5mm). The electrodes were positioned on top of the area closest to the muscles studied (masseter and anterior temporal) parallel to the muscle fibers and with the silver plates placed perpendicularly in relation to them, so as to maximize electrical activity acquisition and minimize noise and interference26. The devices were affixed to the children's skin with electrode adhesives (Stampa®) and 3M Transpore hypoallergenic tape.

### VI. Data analysis

Quantitative data analysis was performed through digital myoelectric signal processing in the domain of amplitude in terms of Root Mean Square (RMS), using software Myosystem. Then the best signal was qualitatively picked for each child from the three data collection sessions done upon the studied muscles. Low noise interference, match between FFT tracing and histograms and myographic records, were the criteria for choosing the best signals.

Three different analyses were carried out in each of the tests (maximal intercuspal position, mastication):

a) Comparison between groups (NB x MB) of average electrical activity found in right and left anterior temporal and right and left masseter muscles.
b) Comparison between groups of the summation of electrical activity for muscles on the right side (right temporal + right masseter) and left side (left temporal + left masseter).
c) Comparison between groups of the summation of electrical activity for temporal muscles (right + left) and masseter muscles (right + left).

Considering the need mentioned in the literature for data normalization to compare electromyographic data and the difficulty inherent to choosing the best way to do it27-30, we decided to analyze the electromyographic data with their values expressed as a percentage.

In order to normalize the data sets, we used the averages captured at maximal intercuspal position for the nasal breathing group for each muscle and test that was carried out. The electrical activity levels recorded for the NB and MB groups were therefore normalized based on the values captured at maximal intercuspal position for the nasal breathing group. Maximal intercuspal position values were equaled to 100% and the percentages for the other values were calculated using simple arithmetic (the rule of threes).

### VII. Statistical method

The data sets were statistically treated using Student's T-test. When the data sets to be compared belonged to different groups, we used the comparison of two averages of non-paired data with unknown population standard deviations. When the comparisons were done within one same group, we used paired data comparison. In order to check standard deviations, although they were unknown and possibly different from one another, before the test mentioned previously test was applied we carried out a Variance Comparison test. We adopted 5% (p<0.05) as a threshold to assign statistical significance to the events studied. All such occurrences were marked with an asterisk in the charts that follow.

## RESULTS

**Chart 1:**
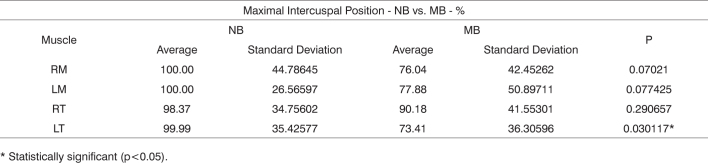
Comparison between normalized averages measured as a percentage of the studied muscles obtained from the nasal (NB) and mouth (MB) breathing groups at maximal intercuspal position.

**Chart 2:**
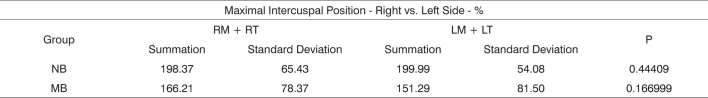
Comparison between summations of normalized electrical activity measured as a percentage of right masseter and anterior temporal muscles (RM+RT) and left masseter and anterior temporal muscles (LM+LT) at maximal intercuspal position for each of the studied groups.

**Chart 3:**
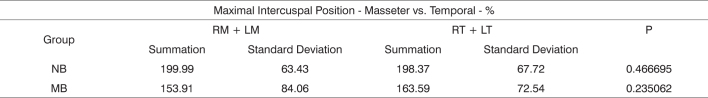
Comparison between summations of normalized electrical activity measured as a percentage of right and left masseter muscles (RM+LM) and right and left anterior temporal muscles (RT+LT) at maximal intercuspal position for each of the studied groups.

**Chart 4:**
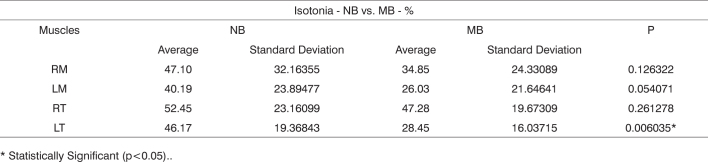
Comparison between summations of normalized electrical activity measured as a percentage of the studied muscles obtained from the nasal breathing (NB) and mouth breathing (MB) groups during isotonia

**Chart 5:**
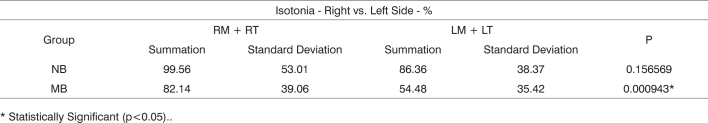
Comparison between summations of normalized electrical activity measured as a percentage of right masseter and anterior temporal muscles (RM+RT) and left masseter and anterior temporal muscles (LM+LT) during isotonia for the studied groups.

**Chart 6:**
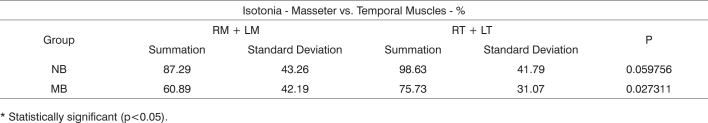
Comparison between summations of normalized electrical activity measured as a percentage of right and left masseter muscles (RM+LM) and right and left anterior temporal muscles (RT+LT) during isotonia for the studied groups.

Results can be found in Chart 1, Chart 2, Chart 3, Chart 4, Chart 5, Chart 6.

* Statistically significant (p<0.05).

* Statistically Significant (p<0.05)..

* Statistically Significant (p<0.05)..

* Statistically significant (p<0.05).

## DISCUSSION

The results found at maximal intercuspal position and usual mastication were discussed together, as both circumstances are related to each other.

Chart 1 shows the normalized electrical activity averages for the studied muscles, collected for groups NB and MB at maximal intercuspal position. Chart 4 presents a similar analysis, however considering usual mastication.

After analyzing the set of results presented in charts 1 and 4, we found that the electrical activity levels were lower in the mouth breathing group for all studied muscles, with statistical significance being however limited to the left anterior temporal muscle.

Among a series of possible alterations found in mouth breathers, hypotonic and hypofunctional jaw elevator muscles and ineffective mastication were observed^10^, ^11^. Mastication may also be affected as a result of jaw elevtor muscle laxity or even by poor coordination between breathing, mastication and swallowing^13^.

The fact that all studied muscles presented lower electrical activity levels among mouth breathers when compared to the nasal breathing group may also be associated with the preference the first have for softer foods^16^, ^31^, which would lead to reduced muscle activity^15^, ^16^, ^32^, ^33^.

Another possible reason for the lower averages of electrical activity in masticatory muscles among mouth breathers when compared to nasal breathing children is the trend towards vertical craniofacial growth found in individuals resorting to mouth breathing^7^, ^8^, ^9^, ^11^, ^23^, that could possibly account for the lower level of electrical activity in masticatory muscles due to the existing relationship between masticatory function and craniofacial development^14^, ^15^, ^17^, ^32^, ^34^. On the other hand, some studies could not find statistically significant differences when comparing electromyographic activity between groups tending to dollic, meso and brachiofacial growth typologies^35^.

The average electrical activity levels found in the studied muscles of mouth breathers indicated the existence of asymmetric muscle activation patterns in this group of patients, as higher levels of activity were identified in the anterior temporal muscles. Such asymmetry and the statistical difference found only in the left anterior temporal muscle may be correlated to the patients’ preferential lateral masticatory pattern - defined in this study through speech clinical evaluation - and altered head posture, often observed among mouth breathers^1^, ^2^, ^3^, ^4^, ^5^, ^6^, ^11^, ^16^, ^18^, ^36^. Not all authors agree with these assumptions, as some tend to assign little clinical relevance to anterior temporal muscle asymmetry, due to the role in stabilization played by this muscle^37^. In the results we found, the increased levels of activity observed in the right temporal muscle when compared to its left counterpart suggests that the first is working in compensatory mode due to the masticatory preferences leaning to the right side among mouth breathers included in our study and the reduced activity observed in the masseter muscles.

When looking at electromyographic and clinical findings together, one may assume that, given the fact that most mouth breathers in this study preferably chew on the right side of their mouths - and that such dynamic task will interfere with muscle static activity in the long run - these individuals have more markedly developed the muscles residing to the side where mastication is more intense. Therefore, both the right masseter and anterior temporal muscles presented higher electrical activity patterns than their counterparts to the left .

Charts 2 and 5 compare the added electrical activity values for the right masseter and temporal muscles against those of the left masseter and temporal muscles for both groups at maximal intercuspal position and during mastication, respectively. By looking at both charts it is possible to realize that, in this group of mouth breathing children, the presented masticatory pattern may be considered more asymmetric than the one presented by nasal breathing children. The right masseter and anterior temporal muscles showed higher levels of electrical activity than their left counterparts, but statistical significance was found only during mastication.

Both in the clinical analysis and electromyographic examination during mastication, most children in the mouth breathing group presented a clear pattern of unilateral mastication. It is believed that when one uses preferentially one side of the mouth to chew, the muscles on that side become more powerful, while their counterparts on the other side become more elongated and with less tone, often times showing a discrete, however perceptible, muscle asymmetry^10^. Thus, even in situations of isometric contraction, represented herein by the test at maximal intercuspal position, such findings may be observed. The asymmetries observed both in static (at maximal intercuspal position) and dynamic (mastication) conditions suggest the presence of common factors impacting both instances^38^.

Likewise, supporting the findings of our study, reports in the literature indicate that functional disorders connected to mouth breathing and ineffective mastication lead to reduced muscle strength and asymmetry associated with unilateral mastication^33^.

According to Chart 3, the nasal breathing group presented a more symmetric muscle activity pattern than that of the mouth breathers, even though the difference was not statistically significant. The mastication test on Chart 6 shows the summations of electrical activity levels of the masseter and temporal muscles for both groups. Mouth breathing children at maximal intercuspal position and during mastication tests presented higher electrical activity levels in their anterior temporal than on their masseter muscles.

According to the literature, the masseter has a more important functional role than the anterior temporal muscle, whose main function is mandibular positioning^15^, ^26^, ^37^, ^39^, ^40^, ^41^, ^42^, ^43^.

Many are the publications describing the interactions between mouth breathing, masticatory, and head and neck muscles^1^, ^17^, ^8^, ^10^, ^18^.

As mentioned previously, a finding common to a multitude of scientific research papers is anterior head posture, craniofacial growth pattern, and preference for softer foods among mouth breathers. As these alterations lead to masticatory dysfunction and they are associated with mouth breathing, it is believed that inadequate respiratory function may have some impact, although not directly, upon mastication.

The results presented in this paper and the references mentioned herein allow for the confirmation of the above described assumption; nonetheless, the determination of causal directions for the identified alterations bring about the controversial discussion on cause vs. effect, thus calling for more specific studies. The increased activity levels found in the anterior temporal muscles at maximal intercuspal position and during mastication (also observed in temporomandibular disorders) of mouth breathing children can be explained by the fact that mouth breathers tend to position their heads anteriorly to facilitate the passing of air, leading to increased electrical activity in the temporal muscles to compensate for the reduced activity levels in the masseter muscles. The results observed in this study also lead to questions that could be addressed by longitudinal research, such as the hypothesis that these children would tend to develop other signs and symptoms of temporomandibular disorder in the future.

Therefore, additional studies are required to enhance the understanding of how mastication alterations manifest themselves in mouth breathers, as well as to determine the most effective treatment for the patients.

## CONCLUSION

When comparing the results obtained for nasal and mouth breathing children, we found that the levels of electrical activity in the masseter and anterior temporal muscles were lower among mouth breathers, with statistical significance however limited only to the left temporal muscle at maximal intercuspal position and during mastication.

Mouth breathers were also found to have increased electrical activity in the right-side muscles during mastication, when compared to the children in the nasal breathing group.

Electrical activity levels in the temporal muscle of mouth breathing children were higher than the levels observed in the masseter muscles during mastication; no difference was found in terms of temporal and masseter muscle electrical activity among the children in the nasal breathing group.

We may therefore conclude that mouth breathing has interfered with the patterns of electrical activity of the anterior temporal and masseter muscles at maximal intercuspal position and during mastication.
